# The Out-Patient

**Published:** 1892-12-24

**Authors:** F. Clarke


### THE OUT-PATIENT.

By Dr. F. Clarke.

Welcome her kindly and tenderly,
Speak to her softly and low;
Though her eye looks bright and her spirit seems light,
Her heart may be breaking below.

Perhaps she is weary, perhaps she is faint,
Or thinly and scantily clad;
Perhaps it is fear of what may be near
Makes her so mournful and sad.

Or perhaps some trial that is sorer still
Is pressing upon her heart,—
Some loved one may lie, sick and ready to die,
And that makes the tear-drops start.

Then bear with her tale of trouble,
Though her speech is broken and slow;
Let her sob, let her weep, her grief is so deep
That her tears they must overflow.

Give heed to all that she has to say
With patient thought and love:
She knows not what to do! she depends upon you
As she stands in her loneliness there.

She has had to leave her wonted work,
She is so anxious and ill,
Trembling and weak, she comes to seek
The help of your knowledge and skill.

Then deal with her gently and lovingly,
Counsel her wisely and well;
The good that you do may be known to few,
But the comfort to *her*—who can tell.

And grudge not time, or toil, or thought;
The reward you shall surely see;
For what you have done to the suffering one,
"You have done it," He saith, "unto Me!"

